# Supplementary material for: BnLPAT2 gene regulates oil accumulation in Brassica napus by modulating linoleic and linolenic acid levels in seeds
Source: PLoS One. 2025 Apr 16;20(4):e0321548. doi: 10.1371/journal.pone.0321548 (PMC12002453; doi:10.1371/journal.pone.0321548)
Supplement: S1 Fig — (DOCX) [file pone.0321548.s008.docx]

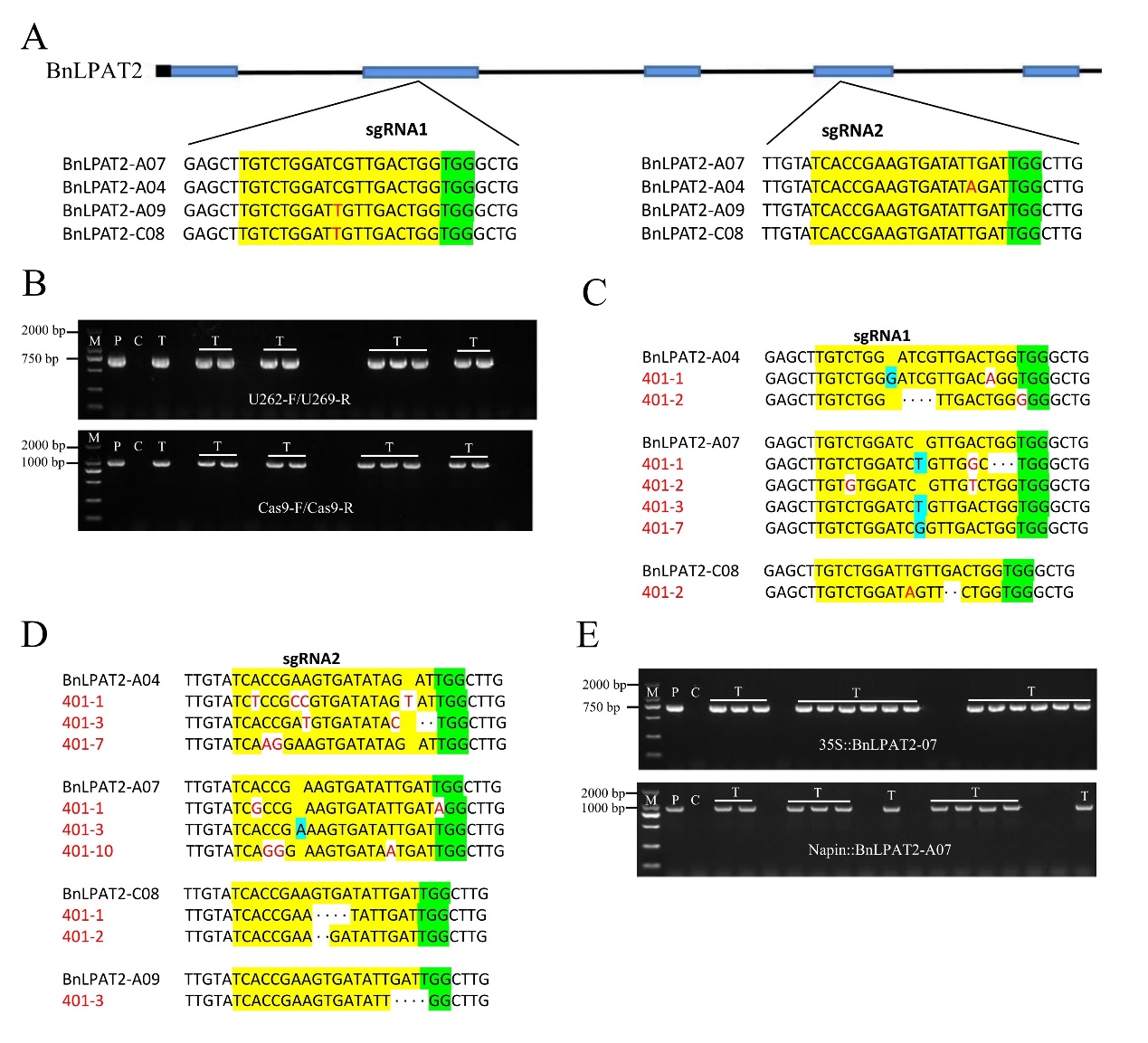


Fig S1. Construction and detection of expression vector.

(A) Sequence comparison of *BnLPAT2* gene homologous copy target site. (B) CRISPR/Cas9 editing rapeseed PCR detection. Primer pair detection diagram of U262-F and U269-R; Cas9-F/R primer pair detection diagram; M: DL 2000 DNA Marker; P: Positive plasmid; W: Wild-type; T: Positive strain. (C and D) Identification of CRISPR/Cas9 Homologous Copy target of *BnLPAT2* gene in T_0_ generation of *B. napus*. (E) PCR detection of transgenic positive overexpressing *B. napus*.
