# Supplementary material for: BnLPAT2 gene regulates oil accumulation in Brassica napus by modulating linoleic and linolenic acid levels in seeds
Source: PLoS One. 2025 Apr 16;20(4):e0321548. doi: 10.1371/journal.pone.0321548 (PMC12002453; doi:10.1371/journal.pone.0321548)
Supplement: S1 Raw Images — (PDF) [file pone.0321548.s009.pdf]

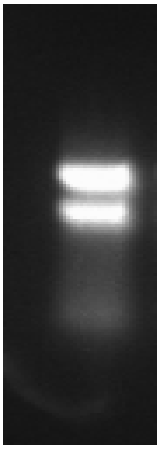

This is the RNA electrophoresis gel image in Fig 1 A.

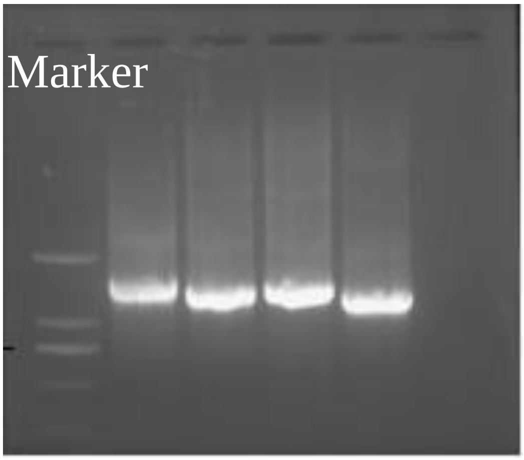

This is the DNA electrophoresis gel image in Fig 1 B, with the marker size of 2000 bp.

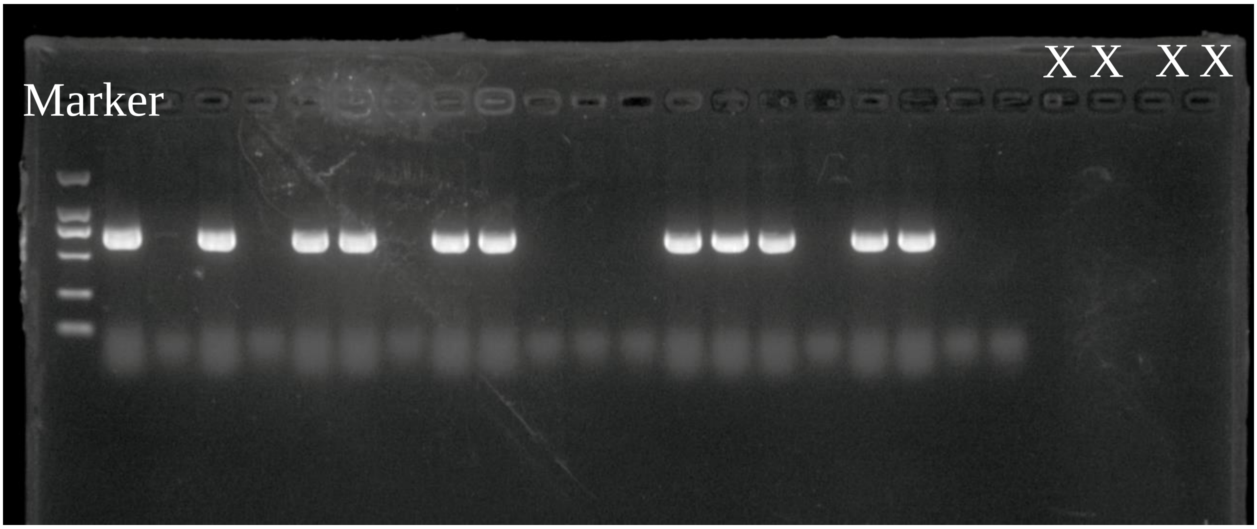

This is the DNA electrophoresis gel image in Supplementary Fig S1 B (U262-F/U269-R), with the marker size of 2000 bp.

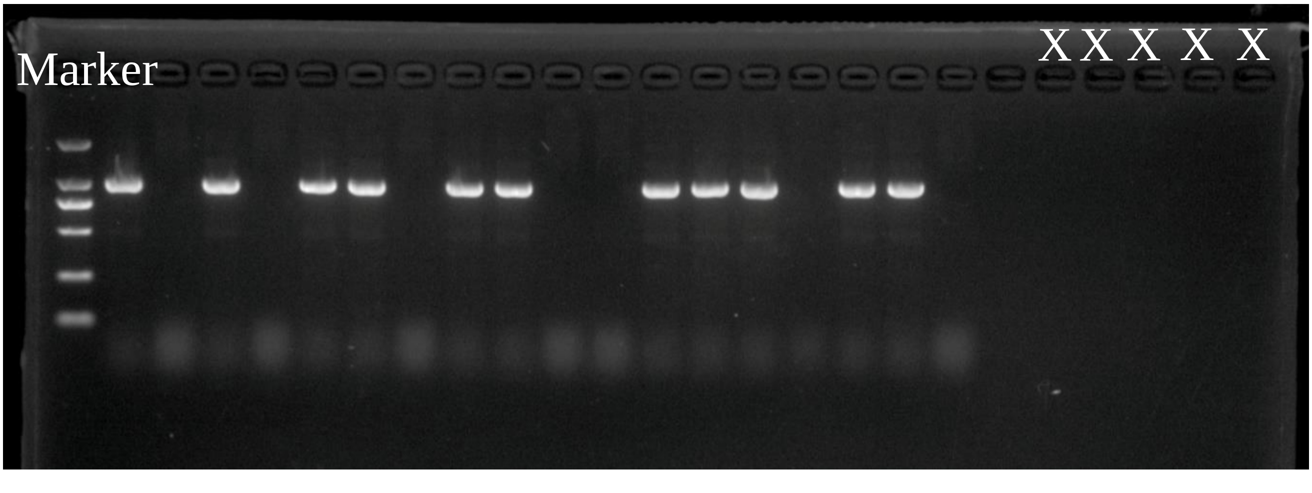

This is the DNA electrophoresis gel image in Supplementary Fig S1 B (Cas9-F/Cas9-R), with the marker size of 2000 bp.

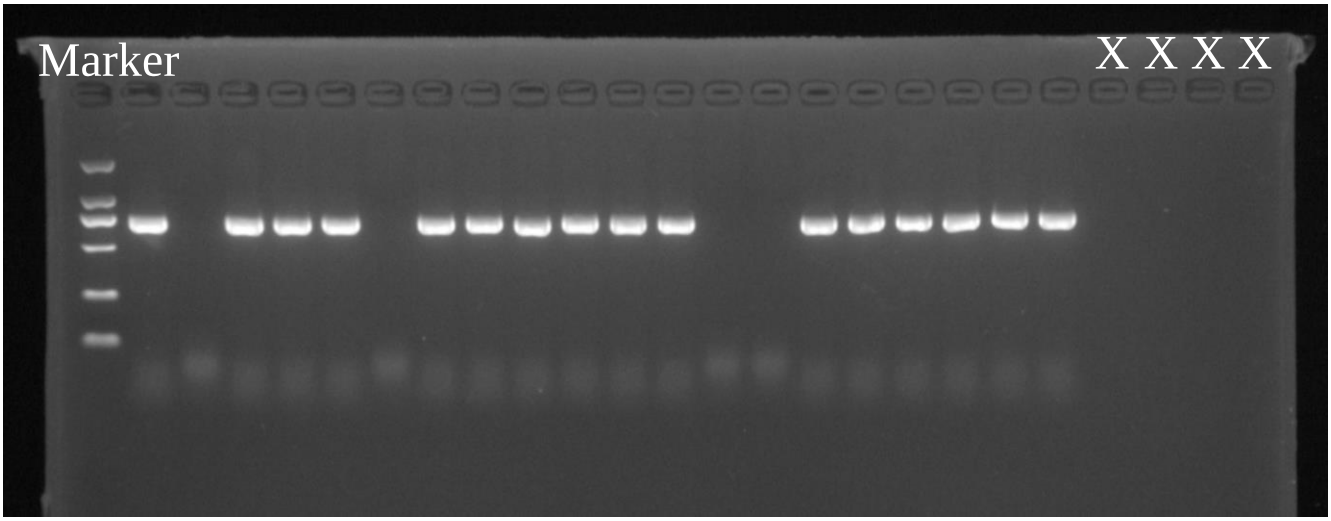

This is the DNA electrophoresis gel image in Supplementary Fig S1 E (35S::BnLPAT2-07), with the marker size of 2000 bp.

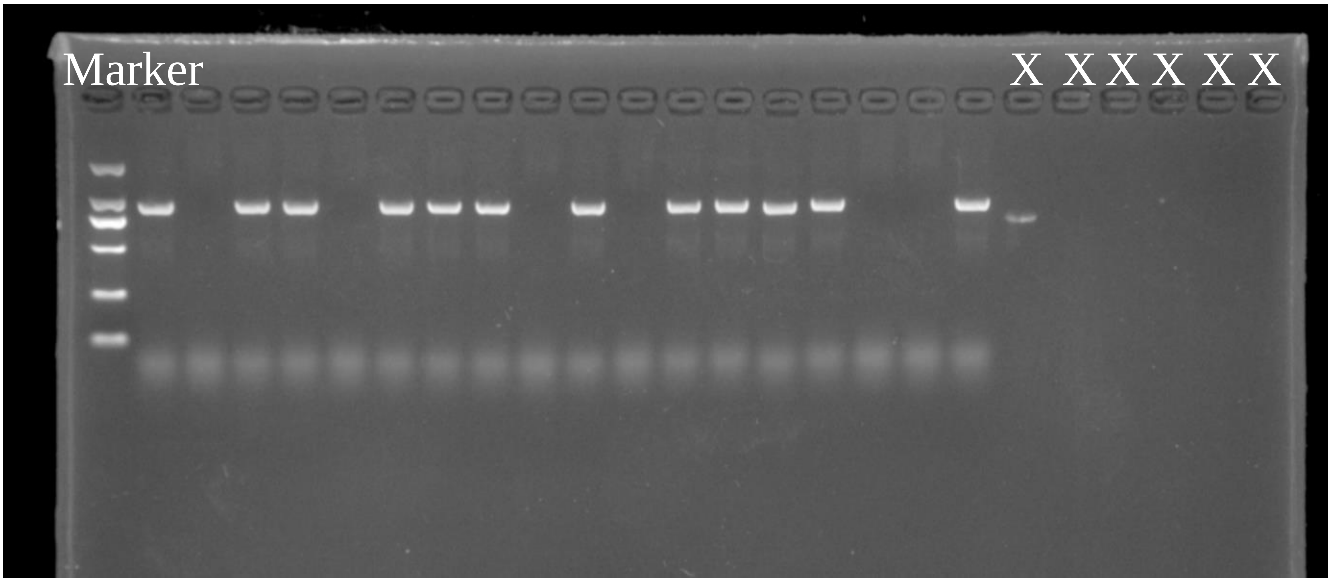

This is the DNA electrophoresis gel image in Supplementary Fig S1 E (Napin::BnLPAT2-A07), with the marker size of 2000 bp.
