# Supplementary material for: BnLPAT2 gene regulates oil accumulation in Brassica napus by modulating linoleic and linolenic acid levels in seeds
Source: PLoS One. 2025 Apr 16;20(4):e0321548. doi: 10.1371/journal.pone.0321548 (PMC12002453; doi:10.1371/journal.pone.0321548)
Supplement: Attachment — Submitted filename: PONE review_6.1.2025.docx [file pone.0321548.s010.docx]

**Manuscript Number:** PONE-D-24-59454
**Manuscript Title:** *BnLPAT2* Gene Regulates Oil Accumulation in *Brassica napus* by Modulating Linoleic and Linolenic Acid Levels in Seeds

**Reviewer:**

Dear editor,

I thoroughly reviewed the manuscript titled "*BnLPAT2* Gene Regulates Oil Accumulation in *Brassica* *napus* by Modulating Linoleic and Linolenic Acid Levels in Seeds." This paper explores the functional analysis of *BnLPAT2* genes using bioinformatics, qRT-PCR, CRISPR/Cas9, overexpression, and transcriptome sequencing. The findings suggest that these genes play a crucial role in regulating plant oil accumulation, optimizing fatty acid composition, and contributing to the synthesis of membrane lipids and signaling molecules. While the paper is well-executed and suitable for publication in PONE, I recommend major revisions before publication to address some necessary corrections and edits. One major concern is the lack of detail in the materials and methods section. I suggest that the authors provide more information about the varieties used, transgenic lines, experimental conditions, and plant growth condition to ensure the reproducibility of the study. Overall, the study presents valuable insights into the role of *BnLPAT2* genes in oil accumulation in *Brassica napus*. With some revisions and additions to the materials and methods section, the paper will be better positioned for publication.

**Abstract:**

- Please include the full names of qRT-PCR and CRISPR/Cas9 in the abstract.

- When selecting keywords, it is important to choose words that are distinct from the title. It is recommended to use a variety of keywords to enhance search engine optimization.

- In the following sentences, 'Overexpression of these genes increased seed oil content and the proportion of C18:2/C18:3 fatty acids, with BnLPAT2-A07 achieving a 6.4% increase in oil content,' the seed oil enhancement should be revised to reflect an increase in seed oil content ranging from 4.46% to 6.44%. This adjustment aligns with the results and discussion section of the study.

**Introduction:**

- In the following sentences, Zhang utilized CRISPR/Cas9 gene-editing technology to conduct targeted editing of multiple homologous copies of BnLPAT2 and BnLPAT5 in rapeseed [27]. Similarly, Chen introduced the peanut *AhLPAT2* gene into Arabidopsis, leading to enhancements in seed weight, oil content, total fatty acids, and unsaturated fatty acids [30]. References should be included after the respective names Zhang [27] and Chen [30].

- In the final paragraph of the introduction, the author should address the gap in previous studies regarding *LPAT2* expression and seed oil accumulation that prompted the current study. The structure of the study's objectives should include a discussion of the issues identified in previous research, the hypothesis being tested in the present study, and the framework of the current investigation. For example, it was hypothesized that *BnLPAT2* may exhibit a preference for linolenic acid and long-chain fatty acids.

**M&M**

How were Transgenic rapeseed plants produced? It is important to clarify the conditions in which the Transgenic rapeseed plants were cultured. Additionally, more details about the genotypes used, such as XY15, ZS6, and ZS11, need to be provided in a cohesive manner throughout the manuscript. It is also essential to include information about the wild type, transgenic lines (T0, T1, and T2), and the conditions under which they were produced. Knockout lines were grown under specific conditions, and the T2 generation was produced for seed collection, oil content analysis, and fatty acid analysis. However, this information was lacking in the Materials and Methods section. It is crucial for authors to thoroughly present all materials, experimental conditions, and procedures so that other researchers can replicate the study with ease and reliability. This will ensure that the protocol can be followed accurately, leading to consistent and reproducible results.

- The determination of seed oil content should be organized into a cohesive paragraph. Additionally, relevant references for Gas Chromatography should be included to support the analysis.

- Please revise the formula for calculating seed oil content (%) as follows:

[(mass of internal standard FA (C17:0) / proportion of internal standard FA (C17:0)] - (mass of internal standard FA (C17:0) / seed mass)

- The section on RNA extraction should include more details, such as the specific tissue part at which phenological stage (time) the extraction is performed, and from which rapeseed varieties (XY15 and ZS6).

- The author collected siliques at various time points, including 7, 14, 21, 28, 35, and 42 days after pollination. However, in the transcriptome analysis, siliques were specifically collected at 30 days after pollination. It is important to understand the rationale behind these different time points and why only 30 days was chosen for the transcriptome analysis. Please provide clarification on this matter.

- Please include the full names of The Gene Ontology (GO) and Kyoto Encyclopedia of Genes and Genomes (KEGG) databases when first mentioned.

**Results**

- Please move these sentences to M&M. Total RNA was extracted from XY15 seeds two weeks after pollination (Figure 1 A). Using reverse transcribed cDNA as a template, PCR amplification produced four target bands, as shown in Figure 1 B. The target fragments were recovered, ligated into the pUCm-T vector, and transformed into DH5α (Escherichia coli). After sequencing and alignment, four full-length CDS sequences were cloned and named BnLPAT2-A04 (1155 bp), BnLPAT2-A07 (1173 bp), BnLPAT2-A09 (1173 bp), and BnLPAT2-C08 (1173 bp), encoding proteins of 384, 390, 390, and 390 amino acids, respectively.

- In this sentence “Secondary structure predictions indicated that BnLPAT2 proteins consisted of random coils”, the phrase "consisted of" should be changed to "consist of".

- In this sentence, it is recommended to include the names of the genes associated with α-helices. The α-helices being the most prevalent, representing 46.15% (BnLPAT2-A04), 47.68% (BnLPAT2-A07), 48.21% (BnLPAT2-A09), and 45.57% (BnLPAT2-C08).

- In the following sentence, 'In contrast, BnLPAT2-A07/A09/C08 share 91.82% and 98.04% similarity with AtLPAT2, respectively (Figure 2 B)', please include the similarity of BnLPAT2-A09 with AtLPAT2. Additionally, what is the level of similarity between the three genes?

- This information 'To analyze the potential functions of the four BnLPAT2 genes in rapeseed, the PlantCARE software was used to analyze the promoter sequences in the 2 kb upstream region of the BnLPAT2 genes' should be include in the Materials and Methods section, as it pertains to the methodology used in the study, rather than the results obtained.

- The number of cis-acting elements in the promoter sequences of the BnLPAT2 genes, categorized in each functional group, should be added.

- Please move the following sentences from the results section to the Materials and Methods section:

“Based on the transcriptome data reported in the BnTIR database (https://yanglab.hzau.edu.cn/BnTIR), the expression levels of six homologous copies of the BnLPAT2 gene across various tissues were analyzed using heatmap analysis (Figure 4 A)”

“Total RNA was extracted from roots, stems, leaves, flowers, and seeds of ZS6 and XY15, and cDNA was synthesized by reverse transcription for qRT-PCR analysis”

- During the RNA extraction process, one location focused on extracting RNA from flower buds, while another location only mentioned flowers. Similarly, one location considered extracting RNA from seeds, while another location referred to different developmental stages of seeds. It is important to ensure consistency and coordination throughout the entire manuscript. Please make the necessary corrections to address these discrepancies.

- The study focused on the expression patterns of BnLPAT2 genes in various tissues of B. napus, specifically examining three genotypes: ZS11, ZS6, and XY15. However, the Materials and Methods section did not mention ZS11 at all. It is important for the author to address this oversight and ensure that all relevant information is included throughout the manuscript for coherence and accuracy.

- Please move the sentence "Seeds from the T2 generation of Arabidopsis were harvested to measure oil content and fatty acid composition" to the Materials and Methods section. Additionally, please provide more detailed information about the T2 generation, including how it was produced and under what conditions.

- Why was the average editing efficiency so low, at less than 17%? Could it be possible that the primer used was not suitable, or that the target site was not highly editable by BnLPAT2-Cas9?

- In the transcriptome analysis section, we initially refer to differentially expressed genes (DEG) and gene ontology (GO) by their full names. Subsequently, we use the abbreviations for DEG and GO. It is important to maintain consistency in terminology throughout the manuscript

- Please provide the full names of hub proteins, including RPS13B, RPL24B, UTP18, RPL29B, RPL35AA, MCM5, and RPS24A. For example, ribosomal protein S13B (RPS13B)...

- These sentences “We searched for the genes encoding these proteins in the BnTIR database and examined their expression in the seeds of *B*. *napus* ZS11” should be relocated to the Materials and Methods section.

- These findings “Recent studies have reported that ribosome synthesis and peptide post-translational modification can form hybrid lipid peptides with fatty acids, playing crucial roles in the fatty acid biosynthetic pathway [36,37]. Although these genes encoding core proteins show high expression in seeds, their involvement in oil synthesis and accumulation in B. napus seeds requires further investigation.” should be discussed in the subsequent section.

**Discussion**

- "In line 14, 'AtLPAT2' should be italicized. Please review the manuscript to ensure that gene names are correctly formatted in italics."

- The cis-acting element in promoter analysis is related to oil content. I suggest the authors discuss this element further, as well as delve into the functional analysis focused on the oil regulatory process.

- Please provide the complete name of "tri-HFA-TAGs"

- The author conducted a study to measure the expression level of *BnLPAT2* during various stages of seed development in siliques (7, 14, 21, 28, 35, and 42 days after pollination). The results showed that the expression of *BnLPAT2* varied at different stages, with higher levels of expression detected only in the mid-to-late stages of seed development, specifically in BnLPAT2-A07. It is important to discuss the differences in expression levels and how they are related to oil accumulation. By understanding the relationship between expression levels of *BnLPAT2* and oil accumulation, we can gain insights into the mechanisms underlying seed development and potentially improve oil production in plants.

- Please include a paragraph discussing the PPI network and hub proteins that play a causal role in oil and fatty acid biosynthesis. It is suggested that we thoroughly analyze each part of the results and its relationship to *BnLPAT2* and oil biosynthesis.

**Conclusion:**

In conclusion, rather than reiterating the results, it is recommended to focus on key points such as the phenological stage at which *BnLPAT2* exhibited higher expression, hub proteins and DEGs associated with *BnLPAT2* and fatty acid biosynthesis, and how *BnLPAT2* enhances oil accumulation. These aspects should be highlighted in the conclusion for a more comprehensive understanding of the study.

**References:**

The genus and species names, as well as the gene names, in the majority of the references are not properly italicized. please double-check references.
