# Supplementary material for: BnLPAT2 gene regulates oil accumulation in Brassica napus by modulating linoleic and linolenic acid levels in seeds
Source: PLoS One. 2025 Apr 16;20(4):e0321548. doi: 10.1371/journal.pone.0321548 (PMC12002453; doi:10.1371/journal.pone.0321548)
Supplement: Attachment — Submitted filename: Response to Reviewers.docx [file pone.0321548.s012.docx]

Dear Reviewer #1,

Thank you very much for your feedback! Your critiques and suggestions have been very helpful to our research. We can see that you have reviewed our manuscript very carefully, which shows that you paid great attention to our work. We greatly appreciate you taking the time to provide us with these valuable suggestions, which will benefit our future research and paper writing. We will carefully revise the manuscript based on your recommendations. Once again, thank you！

Response Abstract: Based on your suggestions, we have added the full names of qRT-PCR and CRISPR/Cas9, included additional keywords, and revised some of the wording in the abstract. We have adjusted the increase in seed oil content to 4.46% to 6.44%, making it consistent with the results and discussion sections.

Response Introduction: We have carefully revised the citation format of the references and added content to the last paragraph of the Introduction as per your suggestion.

Response Abstract: Based on your suggestions, we have added the full names of qRT-PCR and CRISPR/Cas9, included additional keywords, and revised some of the wording in the abstract. We have adjusted the increase in seed oil content to 4.46% to 6.44%, making it consistent with the results and discussion sections.

Response Introduction: We have carefully revised the citation format of the references and added content to the last paragraph of the Introduction as per your suggestion.

Response M&M:

-Our transgenic Arabidopsis and rapeseed were generated by transformation and selection methods referenced in [33][34], so we did not provide detailed methods in this manuscript. Based on your suggestion, we have included more detailed information about the production and cultivation of the transgenic plants. The obtained T0 generation and the selected T1 and T2 plants were all grown in a plant growth chamber at 24°C with a light cycle of 16 h/8 h. The detailed information has been added to the M&M section.

-We have added the method steps for fatty acid and oil content determination by gas chromatography used in this experiment. The details of RNA extraction have also been thoroughly supplemented. We chose seeds from 30 days after pollination for transcriptome data because the period of rapid lipid accumulation in *Brassica napus* seeds occurs between 25 and 35 days. Through qRT-PCR, we found that the expression of the *BnLPAT2* gene in seeds at 7, 14, 21, 28, 35, and 42 days follows a dynamic change process. Therefore, we selected seeds at 30 days, during the period of rapid oil accumulation, for transcriptome sequencing analysis.

-We have included the full names of terms such as Gene Ontology (GO) and Kyoto Encyclopedia of Genes and Genomes (KEGG) databases the first time they are mentioned.

Response Results:

-Based on your suggestions, we have added the names of genes related to α-helix. Regarding your comment about some sentences in the Results section that should be moved to the M&M section, we have carefully revised this and transferred certain sentences from the Results to the M&M section. You can refer to our revised manuscript for specific changes.

-We have clarified in the manuscript that since the amino acid sequences of BnLPAT2-A09 and BnLPAT2-C08 are identical, the sentence "In contrast, BnLPAT2-A07/A09/C08 share 91.82% and 98.04% similarity with AtLPAT2, respectively (Figure 2 B)" already includes the similarity between BnLPAT2-A09 and AtLPAT2. We believe that the comparison of amino acid sequence differences is more important, so we did not display the gene comparison. Additionally, to some extent, amino acid sequence differences reflect gene sequence differences.

-Regarding your comment about the absence of ZS11 in the Materials and Methods section, it is because we did not grow the ZS11 rapeseed; instead, we used gene expression data of ZS11 downloaded from the BnIR database for comparative analysis.

-Our gene editing efficiency in the T0 generation is around 20%. We do not consider this gene editing efficiency to be too low. Of course, our technique might not be perfect, but nevertheless, we successfully obtained the edited plants with the target gene.

-We have added the full names of the proteins RPS13B, RPL24B, UTP18, RPL29B, RPP35AA, MCM5, and RPS24A. Additionally, we have moved the following sentence "Recent studies have reported that ribosome synthesis and peptide post-translational modification can form hybrid lipid peptides with fatty acids, playing crucial roles in the fatty acid biosynthetic pathway [36,37]. Although these genes encoding core proteins show high expression in seeds, their involvement in oil synthesis and accumulation in B. napus seeds requires further investigation." to the Discussion section.

Response Discussion:

-In line 14, "AtLPAT2" refers to the protein, so it is not italicized. We have carefully reviewed the gene names throughout the manuscript and made corrections where necessary.

-Based on your suggestions, we have revised the Discussion, particularly regarding the PPI network and core proteins. We have added a section discussing the role of ribosomal proteins in fatty acid and oil synthesis. Please review this addition in the revised manuscript.

Response References:

-We have properly italicized the species names and gene names in the references.

Dear Reviewer #2,

Thank you very much for your recognition of our research work. Your criticisms and suggestions will further help in the development of our future research. Below are our responses to your comments and suggestions on this study:

Response 1: *Brassica napus* is an allopolyploid species, which typically has multiple gene copies. Our initial research involved searching for the *AtLPAT2* (AT3G57650) in the TAIR database, then transferring the Arabidopsis gene IDs to the *Brassica* *rapa* and *Brassica oleracea* databases. We used homologous alignment to identify the *BnLPAT2* genes in *Brassica napus*. At that time, very few *Brassica napus* genome sequences were published, and ultimately, we were able to successfully clone and identify four *BnLPAT2* genes.

Response 2：That study investigates the role of Lysophosphatidic Acid Acyltransferase (LPAT) enzymes, *BnLPAT2* and *BnLPAT5*, in seed oil accumulation (SOC) in *Brassica napus*. The research demonstrates that overexpressing *BnLPAT2* and *BnLPAT5* significantly increases SOC, while knocking down or knocking out these genes results in a decrease in SOC. *BnLPAT2* enhances diacylglycerol synthesis to increase SOC, while *BnLPAT5* primarily promotes phosphatidic acid (PA) synthesis for membrane lipids. The transcriptome analysis in this article also highlights DEGs related to SOC involved in processes such as fatty acid and long-chain fatty acid biosynthesis. However, the article does not present results or discussions regarding the regulation of fatty acid synthesis by the *BnLPAT2* genes. A large number of reports have shown that the introduction of *LPAT2* into Arabidopsis not only increases seed oil content but also significantly alters the levels of linoleic acid, alpha-linolenic acid, and other fatty acids, although the underlying mechanisms remain unresolved. Therefore, in our article, we present the role of *BnLPAT2* in seed oil synthesis and fatty acid accumulation and provide a detailed discussion of this process.

Response 3: With the development of technology, transcriptome sequencing has become very common. In our article, we presented important transcriptome analysis results, and we are preparing to upload the transcriptome sequencing data to the NCBI database. If needed, you can contact the authors via email, and we will be happy to share our sequencing data.

Response 4: We have reviewed the article based on your suggestion, adding hyperlinks to the TAIR database and italicizing the gene names.

Response 5: We have revised the results and conclusion sections, and the changes can be seen in the revised manuscript.

Response 6: We have added statistical significance annotations below the figure legends.

Response 7: We have made revisions to the language and phrasing of the article.
